# Iliocaval Confluence Stenting for Chronic Venous Obstructions

**DOI:** 10.1007/s00270-015-1068-5

**Published:** 2015-03-14

**Authors:** Rick de Graaf, Mark de Wolf, Anna M. Sailer, Jorinde van Laanen, Cees Wittens, Houman Jalaie

**Affiliations:** 1 Department of Radiology, Maastricht University Medical Centre (MUMC), P. Debyelaan 25, 6229 HX Maastricht, The Netherlands; 2 Department of Surgery, Maastricht University Medical Centre (MUMC), Maastricht, The Netherlands; 3 Department of Surgery, University Hospital Aachen, Aachen, Germany

**Keywords:** Deep venous thrombosis, Inferior vena cava, Iliac vein, Stent

## Abstract

**Purpose:**

Different techniques have been described for stenting of venous obstructions. We report our experience with two different confluence stenting techniques to treat chronic bi-iliocaval obstructions.

**Materials and Methods:**

Between 11/2009 and 08/2014 we treated 40 patients for chronic total bi-iliocaval obstructions. Pre-operative magnetic resonance venography showed bilateral extensive post-thrombotic scarring in common and external iliac veins as well as obstruction of the inferior vena cava (IVC). Stenting of the IVC was performed with large self-expandable stents down to the level of the iliocaval confluence. To bridge the confluence, either self-expandable stents were placed inside the IVC stent (24 patients, SECS group) or high radial force balloon-expandable stents were placed at the same level (16 patients, BECS group). In both cases, bilateral iliac extensions were performed using nitinol stents.

**Results:**

Recanalization was achieved for all patients. In 15 (38 %) patients, a hybrid procedure with endophlebectomy and arteriovenous fistula creation needed to be performed because of significant involvement of inflow vessels below the inguinal ligament. Mean follow-up was 443 ± 438 days (range 7–1683 days). For all patients, primary, assisted-primary, and secondary patency rate at 36 months were 70, 73, and 78 %, respectively. Twelve-month patency rates in the SECS group were 85, 85, and 95 % for primary, assisted-primary, and secondary patency. In the BECS group, primary patency was 100 % during a mean follow-up period of 134 ± 118 (range 29–337) days.

**Conclusion:**

Stenting of chronic bi-iliocaval obstruction shows relatively high patency rates at medium follow-up. Short-term patency seems to favor confluence stenting with balloon-expandable stents.

## Introduction

Recanalization and stenting of chronic deep venous obstructions is a minimally invasive intervention that has proven effective and safe over the last decades [1]. With low complication rates and substantial clinical improvement, this procedure has been increasingly performed over the last years. Long-term stent patency rates however vary substantially between clinical reports. Although encouraging 92 % stent patency after 10 years has been reported by dedicated high-volume centers [2, 3], lower results were published by clinics with lesser numbers of patients [4, 5]. Partly, this variation might be explained by lack in dedicated venous interventional tools, e.g., stents. However, in recent years, a lot has changed both in interventional materials and techniques, which might be vital to achieve an optimal result. Therefore, it is of great importance to report on experience with new developments in stent design and stenting technology to help establish an optimal protocol, both for defining the right indication and choosing the optimal treatment.

Chronic vena cava obstruction can stay asymptomatic for a long time. Extensive collateralization through paralumbar veins and the azygos system might obscure the underlying hemodynamic severity for many years into adulthood. However, many patients will be confronted with decompensation at some point in their life, which becomes clinically evident by acute, and in many cases bilateral deep venous thrombosis (DVT). Reconstruction of the occluded inferior vena cava (IVC) by means of angioplasty and stent placement has been performed previously with satisfactory to excellent results [6–8]. However, stenting of the common iliac vein confluence has only been thoroughly described by Neglén et al. [9]. In their study, different stenting techniques were suggested to reconstruct the iliac confluence, all with their own limitations. In this article, we describe our experience with two confluence stenting techniques, one with merely self-expandable stents, the other with balloon-expandable stents bridging the common iliac vein confluence.

## Materials and Methods

From November 2009 to August 2014, 741 patients were evaluated for chronic venous insufficiency/post-thrombotic syndrome (CVI/PTS) or acute central venous obstructive complaints at our tertiary medical center. Forty of these patients were diagnosed with chronic iliocaval obstruction. Chronic iliocaval obstruction was defined as 50 % or more venous lumen obstruction [10] of the IVC involving the confluence on Duplex ultrasound (DUS) or magnetic resonance venography (MRV) with presence of post-thrombotic trabecular changes in both common iliac and external arteries. All patients received DUS and MRV before treatment and cases were discussed in a multidisciplinary team consisting of a venous vascular surgeon, interventional radiologists, a dedicated ultrasonographer and experienced nurses.

All procedures were performed under general anesthesia and full anticoagulation. Patients were administered at least one dose of 5000 IU of heparin during the procedure. After the intervention, patients received a single dose of Low-Molecular-Weight Heparin (LMWH, Tinzaparine) and the pre-intervention anticoagulation regiment was continued, in general aiming for an international normalized ratio (INR) of 3.0–4.0. Patients who did not receive pre-interventional anticoagulation were started on coumarins directly post-stenting aiming for the same INR range, they received daily LMWH’s until this INR range was achieved (with a minimal treatment time of at least 5 days). When no underlying thrombophilia was proven, anticoagulation therapy was continued for at least 6 months.

### Stenting Technique

**Fig. 1:**
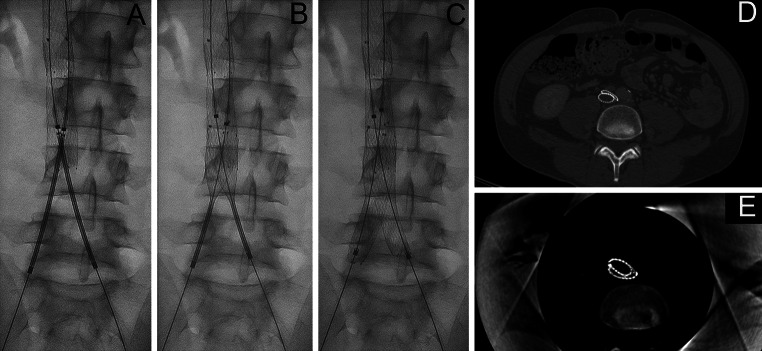
Confluence stenting with self-expandable stents (SECS). **A**–**C** self-expandable stents are placed within the large diameter IVC stent, extending into the common and/or external iliac veins. **D** CT shows compression of one iliac self-expandable stent, while the adjacent stent shows adequate expansion. **E** per-operative conebeam CT shows another example of significant compression by the contralateral stent

Patients with IVC and bilateral iliac vein obstruction and debilitating complaints interfering with daily life (most often chronic pain and/or venous claudication), with or without skin changes or ulceration (CEAP C4–6) were offered stent placement. Patients with IVC and bilateral iliac vein obstruction and acute bilateral DVT were also indicated for stenting. Procedures were performed in a dedicated angio suite or hybrid OR. Venous access was obtained through the femoral vein, at least 15 cm distally to the deep femoral inflow under ultrasound guidance. This access site was chosen to obtain optimal angiographic evaluation about the inflow from the deep femoral vein and the collateral network as well as to provide a sufficient distance to the landing zone for stenting. The technique for traversing stenotic and occluded venous segments has been described before [11]. After successful recanalization thorough pre-dilation was done up to 25 mm for the IVC and 14–16 mm for the external and common iliac vein. Due to the elasticity of the lesions, all pathologic venous segments that are being pre-dilated should generally be stented. Based upon the design of the stents used to treat diseased vein segments, we gained experience with two stenting protocols. In our earlier experience, stenting was performed with nitinol self-expandable stents to treat the entire diseased venous segments from the vena cava down to the external iliac vein (SECS group; Fig. 1). These self-expandable stents (sinus XL, Optimed®, Ettlingen, Germany) are registered for treatment of vena cava obstructions, because of the large diameters available and their high crush resistance. In all cases, the vena cava was stented with a diameter of 24 mm. Bilateral extensions were performed with 16 mm nitinol stents (sinus XL, sinus Venous and sinus XL-flex, Optimed®, Ettlingen, Germany and Zilver Vena (Cook®, Galway, Ireland), which were placed parallel inside the vena cava stent with a 2 cm overlap (Fig. 1).

**Fig. 2:**
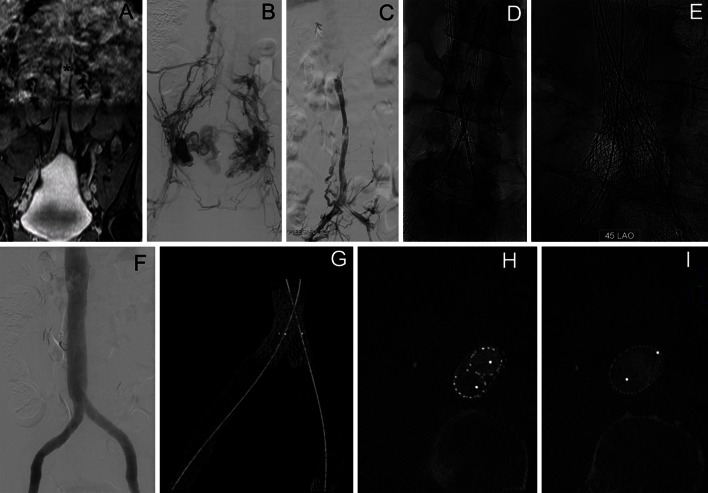
Confluence stenting with balloon-expandable stents (BECS). **A** pre-operative MR venography shows an IVC remnant (*arrow*). Notice the post-thrombotic scarring in the external iliac vein (*arrowhead*). *Asterisk* aorta. **B** angiography shows severe obstruction of bilateral iliac veins and no filling of the IVC. **C** after iliac recanalization angiography from the level of the confluence shows a long stenosis in of the IVC. **D** and **E** positioning and deployment of two balloon-expandable stents. **F** completion angiography shows excellent flow from both iliac limbs through the newly constructed iliac confluence and IVC. **G**–**I** Conebeam CT reconstructions showing perfect positioning and expansion of the stents at the confluence and IVC

In order to minimize stent strut interactions at the confluence, an alternative technique was used where two balloon-expandable stents (AndraStent, Andramed®, Reutlingen, Germany) were placed in the vena cava stent toward the common iliac veins (BECS group; Fig. 2). Bilateral iliac extensions were then performed with 16 mm nitinol stents (sinus Venous® or Zilver Vena stents®). In both groups, post-dilation of the entire stented segments was always performed using a balloon with same diameter as the stents implanted. Inflation time was kept to a minimum, with the sole purpose to let the stents deploy completely. In cases of incomplete deployment, high pressure balloons were used to reach full deployment. Post-stenting angiography was done by means of Conebeam CT imaging (AlluraClarity, Philips Healthcare, Eindhoven, The Netherlands) to evaluate stent expansion and apposition (Fig. 2).

### Endophlebectomy and arteriovenous fistula creation

Extension of post-thrombotic changes at the level of the femoral vein inflow was seen in 15 (38 %) patients. The decision to perform adjunctive surgical desobstruction was pre-operatively made based on MRV imaging. When trabeculations were visible in the common femoral vein extending to both the femoral and the deep femoral (profunda) vein, peripheral venous inflow was deemed insufficient. Use of MRV imaging for evaluation of severity and extent of the disease (trabeculations) proofed consistent during surgery meaning desobstruction was performed in all the selected cases. In these 15 patients, a hybrid procedure consisting of endovascular recanalization and stenting combined with surgical groin incision, endophlebectomy, and creation of an arteriovenous fistula (AVF) was performed to optimize inflow and secure short-term stent patency. In the other 25 patients where no post-thrombotic changes at level of the deep femoral vein inflow were seen, merely recanalization and stenting was performed.

Post-operative stent patency was evaluated by DUS. All patients received follow-up according to a strict protocol consisting of DUS imaging 1 day post-intervention, 2 and 6 weeks after the intervention as well as 3, 6, and 12 months after the intervention and annually thereafter, or if recurrence of symptoms required earlier evaluation. Patency was defined as patency of more than 50 % of the venous lumen on DUS. Direct post-interventional as well as 2 and 6 week DUS examination enabled us to perform timely catheter-directed thrombolysis in case of acute re-occlusion.

### Statistical Analyses

Categorical data are presented by frequencies and percentages. Continuous data are expressed by mean or median values with range and standard deviation. Kaplan–Meier survival analysis was used to calculate patency rates per extremity for all patients (80 legs) as well as for the endovascular only group (50 legs); (Graphpad prism version 5.00 for Microsoft Windows, Graphpad Software. San Diego, CA). Survival percentages with a standard error of the mean (SEM) of >10 % were discarded as being unreliable.

## Results

### Patient Demographics

The patient group consisted of 24 female and 16 male with a mean age of 41 ± 14.9 years (range 16–62 years). MRV determined IVC obstruction with complete certainty in 100 % of cases. In all cases, intraluminal changes, i.e., trabeculations were seen, suggestive of post-thrombotic fibrosis due to earlier DVT. In 6 (15 %) patients, no clinically obvious DVT was noted in their medical history, but the combination of DUS and MRV showed post-thrombotic changes in the venous system in all of these patients. Patients were treated on average 12 ± 8.5 years (range 2–27) after their first DVT. Clinical signs, as scored in the CEAP classification, are shown in Table 1.

**Table 1:** Overview of CEAP clinical classification [16] for the study population (40 right and 40 left legs)

CEAP C	Right leg		Left leg	
Class	(*n*)	(%)	(*n*)	(%)
C0	1	2.5	3	7.5
C1	5	12.5	5	12.5
C2	7	17.5	7	17.5
C3	11	27.5	10	25
C4	9	22.5	9	22.5
C5	4	10	4	10
C6	3	7.5	2	5

Acute bilateral DVT was the indication for treatment in 6 (15 %) patients, skin changes and ulceration in 18 (45 %) patients, venous claudication in 15 (38 %) patients, and chronic lower extremity pain without claudication in one (3 %) patient.

### Interventions

All 40 patients with iliocaval obstructions were recanalized successfully. The average length of vena cava occlusion was 13.8 (range 5–21) cm. The average total length of iliocaval obstruction was 31.2 cm (range 24–37). No per-procedural bleedings or other complications were encountered. In 24 patients, self-expandable stents were used to construct the confluence (SECS group), while in 16 patients a combination of balloon-expandable and self-expandable stents was used (BECS group).

### Follow-up

**Fig. 3:**
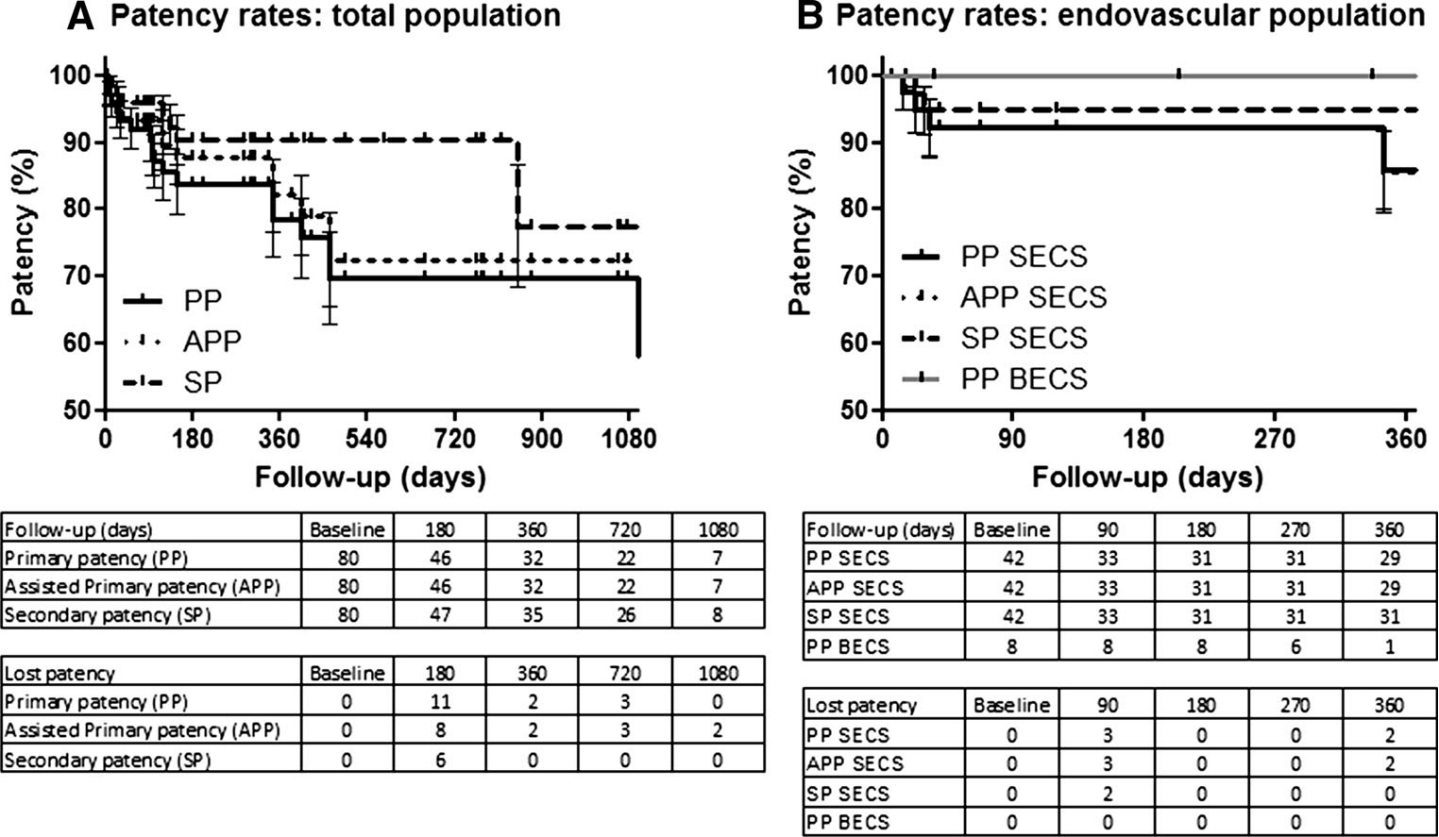
Kaplan–Meier analyses of primary and secondary patency of lower extremities in the total population (80 legs) as well as in the subpopulation of cases treated by endovascular means only (50 legs)

Mean time until latest DUS follow-up examination was 443 ± 438 days (range 7–1683 days). In 33 (82 %) patients, the vena cava and both iliac veins were patent at latest follow-up. In four (10 %) of the patients with stent re-occlusions, one of the iliac veins was occluded at latest follow-up, while the contralateral side and vena cava remained patent. One patient showed total occlusion of the stented area, however clinical signs remained mild during follow-up, possibly because of a well-developed collateral network, re-intervention was therefore postponed indefinitely. Moreover, secondary patency after re-occlusion was obtained by sole thrombolysis in three and catheter-directed thrombolysis combined with re-stenting in another three patients (in one also an AVF was created), in one patient a deep venous bypass was created surgically. This leads to patency rates of treated lower extremities at 12 months of 79, 82, and 90 % (SEM 5.6, 5.5, and 3.8), and at three years of 70, 73, and 78 % (SEM 6.9, 7.1, and 9.0), for primary, assisted-primary, and secondary patency, respectively (Fig. 3A).

Specific analysis of the population treated by endovascular means only (without endophlebectomy and AV-fistula creation) shows higher patency rates. Twelve-month patency rates in the SECS group were 85, 85, and 95 % for primary, assisted-primary, and secondary patency, respectively (Fig. 3B). In the BECS group, primary patency was 100 % during a mean follow-up period of 134 ± 118 (range 29–337) days (Fig. 3B).

Observed complications did not lead to long-term impairment (Table 2). Although re-occlusions occurred, no patients reported worsening of their symptoms.

**Table 2:** Overview and quantification of complications observed in 40 patients treated with caval-bi-iliac recanalization and stenting

Complication	Frequency	%
Reocclusion in one or both iliac veins or IVC	10^a^	25.0
Significant stenosis due to AVF	4	10.0
Major bleeding	3	7.5
Minor bleeding	3	7.5
Lymph leakage/lymphocele	3	7.5
Stenosis deemed non-hemodynamically significant	7	17.5
Residual compression	3	7.5
Tapering	5	12.5
Stent kinking	1	2.5
Stent fracture	1	2.5

^a^Six patients treated by catheter directed thrombolysis

## Discussion

The etiology of IVC obstruction has not yet been determined completely. A congenital origin has been proposed [12]. Central line placement and subsequent thrombosis in premature life might be another explanation [13]. In the present study population, only two patients had endured such an intervention. Sudden IVC thrombosis in later life might develop in case of compression by benign or malignant masses or adjacent anatomic structures. However, we did not see such underlying pathology in our patient population. True absence of the IVC has also not been noticed in our group of 741 patients evaluated for signs of deep venous obstruction between 2009 and 2014. Although this might be suspected on angiography because no contrast dye is seen in the extent of the IVC, MRV always showed a visible remnant (Fig. 2A–C).

Iliac vein and IVC stenting has been performed successfully since more than a decade, has proven to be effective and is considered the first line of treatment in chronic venous obstructive disease [14]. Nevertheless, experience was limited to a small number of centers for many years, with the group of Neglén et al. reporting on the largest number of patients by far [3, 9, 15]. Recently, recanalization and stenting of chronic venous obstructions became more widespread and probably will increase to mature in years to come. Although the endovenous approach has been suggested straightforward, innovative materials enable us to explore alternative approaches, which might reduce complications and improve results. Specifically, for confluence stenting, only few techniques and stent configurations have been proposed, mainly due to limitations in stent design. In the vast majority, the Wallstent™ (Boston Scientific®) was selected as the stent of choice, which limited stent configuration options. However, with the introduction of nitinol and cobalt–chromium stents appropriate for use in the venous system, other techniques have become available. We discuss our experience and patency results with these stents to treat bilateral iliocaval obstruction by means of confluence stenting.

We used two different techniques to construct the new confluence. In our earlier cases, we placed two self-expandable stents within a wide-diameter stent positioned in the distal vena cava just above the confluence, which were extended distally as far as required. With the help of per-procedural rotational angiography and coincidental CT examinations, it was noted though that stent compression was a main issue (Fig. 1). When two self-expandable stents are placed within a restricted space, one is likely to become greater in diameter than the other one, thereby compressing the contralateral side which is corroborated in the diminished short-term patency in the SECS group. Thus, we decided to optimize our protocol by bridging the confluence with two ultra-strong parallel positioned balloon-expandable stents. The BECS group showed a 100 % short-term primary patency. Although the present confluence technique has been mentioned before, no clear description of the technique and interventional tools were given. Furthermore, specific outcome data were not discussed [6].

**Fig. 4:**
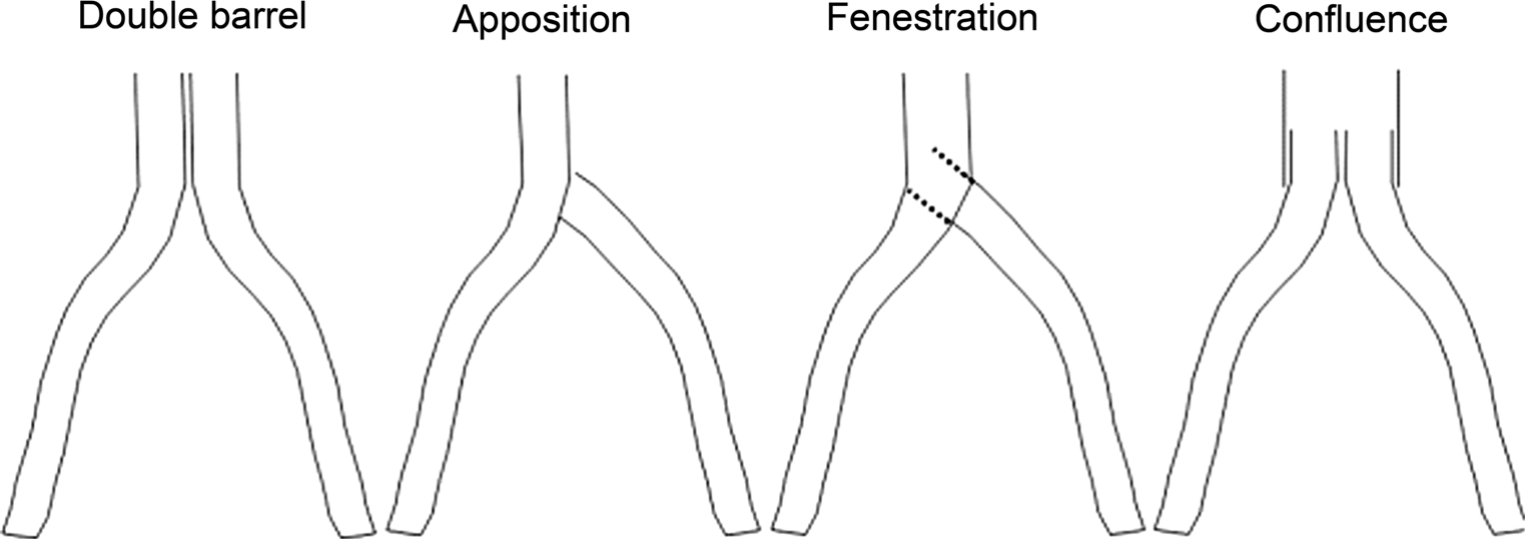
Techniques to treat caval-bi-iliac venous obstruction. The double barrel technique is performed by placing two parallel stents into the IVC as an extension from both iliac veins. In the apposition technique, the ipsilateral iliac stent is extended into the IVC while the contralateral iliac stent is placed in close contact to the former. In the fenestration technique, the ipsilateral stent is pierced and the contralateral limb is then maneuvred through this fenestration. The confluence technique is performed by large diameter stents in the IVC. With a small overlap, two balloon-expandable or self-expandable stents are used as an extension into both iliac veins

Other stenting techniques to treat the common iliac vein confluence have been described earlier [9]. Neglén et al. evaluated three techniques to perform bilateral stenting at the iliocaval confluence (Fig. 4). In our opinion, both the fenestration and apposition technique should be considered inferior to the confluence technique. The fenestration technique requires purposeful fracturing of the contralateral iliac stent, which increases the risk of complications like balloon rupture and subsequent entrapment of devices. Furthermore, re-interventions like thrombectomy might be hampered, which is also true for the apposition technique. Confluence stenting with balloon-expandable stents helps overcome potential problems related to re-interventions. Finally, recreation of the iliac confluence generates flow dynamics that might approach the physiological situation best. With the use of dedicated stents, we introduced an alternative technique to create an efficient and safe true confluence configuration (Figs. 2, 4). Based upon our experience, we suggest the BECS technique to be superior to treat chronic bilateral iliocaval obstructions. It should however be noted that the BECS technique might not be the best option in women at child bearing age, since these stents may non-reversibly compress during pregnancy or birth.

There are some limitations to our study. First, the observational design of the study makes it difficult to draw strong conclusions toward the optimal confluence stent technique. However, availability of stents on the market precluded the possibility to perform a randomized design of the study. Nevertheless, our experience adds to the knowledge of venous stenting thus far reported and might aid in the process of product development and treatment protocols. Secondly, we used different stents to make peripheral extensions from the confluence toward the groin. In the beginning, we used nitinol closed-cell stents in the iliac veins. Since 2013, “dedicated venous stents” were used. “Dedicated” means that the stent manufacturer implies that the stent is better accommodated to the venous anatomy. Usually, these stents are larger in diameter, show good flexibility and high hoop force. Although these venous stents are supposed to perform better in the venous vasculature, up to now there is no proof that these stents improve outcome. Therefore, we have to conclude that the differences in patency between the SECS and BECS group are more likely based upon the configuration of confluence stenting. Based upon our experience with stenting across the common iliac vein confluence, we suggest preferable stenting technique to treat bilateral iliocaval obstructions. Preservation of bilateral symmetric inflow, protection of stent integrity, and avoidance of abundant stent material at the level of the confluence suggest that the BECS technique is superior to earlier described techniques. Evolution however is continuing, and we await the development of “confluence-stent” devices that have already been recognized in bifurcated aorta repair and might also support venous reconstructions.
